# *KCNQ2* mutations in childhood nonlesional epilepsy: Variable phenotypes and a novel mutation in a case series

**DOI:** 10.1002/mgg3.816

**Published:** 2019-06-14

**Authors:** Inn‐Chi Lee, Tung‐Ming Chang, Jao‐Shwann Liang, Shuan‐Yow Li

**Affiliations:** ^1^ Division of Pediatric Neurology, Department of Pediatrics Chung Shan Medical University Hospital Taichung Taiwan; ^2^ Institute of Medicine, School of Medicine Chung Shan Medical University Taichung Taiwan; ^3^ Department of Pediatric Neurology Changhua Christian Children's Hospital Changhua Taiwan; ^4^ Graduate Institute of Medicine Kaohsiung Medical University Kaohsiung Taiwan; ^5^ Department of Pediatrics Far Eastern Memorial Hospital New Taipei City Taiwan; ^6^ Genetics Laboratory and Department of Biomedical Sciences Chung Shan Medical University Taichung Taiwan

**Keywords:** childhood epilepsy, epileptic encephalopathy, *KCNQ2*, phenotypes

## Abstract

**Background:**

Epilepsy caused by a *KCNQ2* gene mutation usually manifests as neonatal seizures during the first week of life. The genotypes and phenotypes of *KCNQ2* mutations are noteworthy.

**Methods:**

The *KCNQ2* sequencings done were selected from 131 nonconsanguineous pediatric epileptic patients (age range: 2 days to 18 years) with nonlesional epilepsy.

**Results:**

Seven (5%) index patients had verified *KCNQ2* mutations: c.387+1 G>T (splicing), c.1741 C>T (p.Arg581*), c.740 C>T p.(Ser247Leu), c.853 C>A p.(Pro285Thr), c.860 C>T p.(Thr287Ile), c.1294 C>T p.(Arg432Cys), and c.1627 G>A p.(Val543Met). We found, after their paternity had been confirmed, that three patients had de novo p.(Ser247Leu), p.(Pro285Thr), and p.(Thr287Ile) mutations and neonatal‐onset epileptic encephalopathy; however, their frequent seizures remitted after they turned 6 months old. Those with the c.387+1G>T (splicing), (p.Arg581*), and p.(Val543Met) mutations presented with benign familial neonatal convulsions. In addition to their relatives, 14 patients had documented *KCNQ2* mutations, and 12 (86%) had neonatal seizures. The seizures of all five patients treated with oxcarbazepine remitted.

**Conclusion:**

*KCNQ2‐*related epilepsy led to varied outcomes (from benign to severe) in our patients. *KCNQ2* mutations accounted for 13% of patients with seizure onset before 2 months old in our study. *KCNQ2* mutations can cause different phenotypes in children. p.(Pro 285Thr) is a novel mutation, and the p.(Pro 285Thr), p.(Ser247Leu), and p.(Thr287Ile) variants can cause neonatal‐onset epileptic encephalopathy.

## 1 INTRODUCTION

*KCNQ2* (OMIM 602235) mutations can contribute to benign familial neonatal convulsions (BFNC) (Biervert et al., 1998; Leppert et al., 1989; Neubauer et al., 2008), benign familial neonatal‐infantile seizures (BFNIS), benign familial infantile seizures (BFIS), and neonatal‐onset epileptic encephalopathy (EE) (Kato et al., 2013; Weckhuysen et al., 2013, 2012). The mutant gene, *KCNQ2*, a voltage‐gated potassium‐channel gene at 20q13, is usually inherited with autosomal‐dominant form in a benign epileptic syndrome associated with a *KCNQ2* mutation. Patients with BFNC, BFNIS, or BFIS usually have seizures as neonates and infants, but they are predicted to have benign outcomes (Leppert et al., 1989; Neubauer et al., 2008; Singh et al., 1998). Most seizures will spontaneously disappear during an infant's first 12 months of life (Coppola et al., 2003; Singh et al., 1998). In *KCNQ2* mutation‐associated neonatal‐onset EE, most mutations are de novo or mosaic inherited, and patients present with severe seizures and severe neurological outcomes (Kato et al., 2013; Weckhuysen et al., 2012). Electroencephalograms (EEGs) in neonatal‐onset EE patients show interictal burst‐suppression or multiple focal spikes (Kato et al., 2013; Weckhuysen et al., 2012). Patients usually have intellectual developmental delays despite seizure remission. A loss (Maljevic et al., 2011; Maljevic, Wuttke, & Lerche, 2008; Wuttke et al., 2008) or gain (Miceli et al., 2015; Millichap et al., 2017) of *KCNQ2* gene function is presumed to be the major mechanism for *KCNQ2*‐associated neonatal‐onset EE. Recent analyses of data from genome‐wide association studies (GWASs) of humans and animals indicate that *KCNQ2* mutations contribute to schizophrenia susceptibility (Choi et al., 2018; Lee, Kim, & Song, 2013). However, outcomes for patients with *KCNQ2* mutations cannot be accurately predicted.

The *KCNQ2* gene is expressed predominantly in the brain and encodes for voltage‐gated potassium‐channel subunits that underlie the M‐current, a repolarizing current that limits repetitive firing during long‐lasting depolarizing inputs (Cooper, Harrington, Jan, & Jan, 2001; Cooper & Jan, 2003; Coppola et al., 2003; Wang et al., 1998). Each subunit of *KCNQ2* consists of heteromultimeric channels with six transmembrane domains (S1–S6): voltage sensors in S1–S4, a loop between S5 and S6 that builds the ion channel pore domain, and a long C‐terminal region of mostly unknown function (Biervert et al., 1998; Cooper et al., 2001; Lerche et al., 1999). The C‐terminal tail contains two helical domains (A and B) that bind to calmodulin (CaM), a calcium (Ca^2+^) sensor (Ambrosino et al., 2015). Helix A contains the consensus CaM binding IQ motif, and helix B mediates Ca^2+^‐dependent CaM binding (Ambrosino et al., 2015; Liu & Devaux, 2014; Zhou et al., 2016). CaM accounts for trafficking protein to cell‐surface membranes. The mutations in the CaM domain have been reported to impair the interaction with calmodulin molecules, and to impair surface expression of potassium channel, which increased action potential firing and hyperexcitability (Maljevic et al., 2008; Zhou et al., 2016).

The precise percentage of neonates and children with *KCNQ2‐*associated epilepsy is unknown. About 163 (1.9%) of the 8,565 patients in one study (Lindy et al., 2018) with epilepsy and neurodevelopmental disorders had detectable *KCNQ2* mutations. Weckhuysen et al. (2013) reported 11 (13%) *KCNQ2*‐associated neonatal‐onset seizures in 84 patients with neonatal‐onset EE. Kato et al. (2013) identified 12 (5%) *KCNQ2*‐associated cases of neonatal‐onset EE in 239 patients. The percentages of *KCNQ2‐*associated epilepsy were not consistent but depended upon what kinds of patients were enrolled. For neonates, rapidly diagnosing and promptly stopping seizures should improve the patient's outcome (Chen et al., 2018; Grinton et al., 2015). The diagnosis can be supported by clinical features, EEG findings, age seizure onset, and family history, and it can be confirmed using a genetic study.

Despite some case‐series reports (Grinton et al., 2015; Kato et al., 2013; Weckhuysen et al., 2013, 2012) and some functional studies (Maljevic et al., 2011, 2008; Wuttke et al., 2008), however, the phenotypes and genotypes are still complex and noteworthy. We previously reported (Lee, Yang, Liang, Chang, & Li, 2017) in a functional study of HEK293 cells that genotype is a major determinant of phenotype. Additional investigations are warranted. In the present study, we investigated a series of cases with *KCNQ2* mutation variants from patients with childhood nonlesional epilepsy.

## 2 MATERIALS AND METHODS

### 2.1 Ethical compliance

Ethical approval of the study was provided by Chung Shan Medical University Hospital's Internal Review Board (IRB #: CS13036).

### 2.2 Recruiting participants

One hundred and thirty‐one patients met all three criteria for “childhood epilepsy without an identified cause” ([1] first seizure when <18 years old, [2] age at last visit <18 years old, and [3] at least one magnetic resonance image [MRI] with no detectable seizure‐related lesions) and were enrolled in the study. Seizure onset occurred before 2 months old for 45 (34%) patients, and between 2 months and 18 years old for the other 86 (66%). The *KCNQ2* genes were sequenced and screened in all patients. If mutations were detected, we requested to sequence and screen the patients’ relatives. Fifty‐five healthy adults who said that they had never had epileptic seizures were enrolled as controls.

### 2.3 Extracting and amplifying DNA from *KCNQ2* exons using a polymerase chain reaction

A genomic DNA purification kit (Gentra Systems; http://www.gentra.com) was used to extract a genomic DNA sample from a peripheral whole blood sample from each patient after we obtained informed consents. All 17 exons of the *KCNQ2* gene were amplified using a polymerase chain reaction (PCR) for each patient.

### 2.4 Purifying and sequencing PCR products

The PCR products were then purified (PCR‐M Clean‐Up System; Viogene‐Biotek Corp., New Taipei City, Taiwan), and their concentrations were measured using a spectrophotometer (Ultrospec 3100 Pro; Amersham Biosciences UK, Little Chalfont, Buckinghamshire, UK). The products were sequenced using an automated DNA sequencer (3100; Applied Biosystems, Foster City, CA). DNA sequencing was done using a kit (ABI PRISM BigDye Terminator Cycle Sequencing Ready Reaction Kit, v3.1; Applied Biosystems) on the ABI PRISM 3730XL DNA analyzer. The sequence data of each patient were checked against the GenBank reference sequence and version number of *KCNQ2* gene (NM_172107.3). Each mutation was numbered and described based on the Mutation Database Initiative (MDI)/Human Genome Variation Society (HGVS) Mutation Nomenclature Recommendations (http://www.hgvs.org/mutnomen or http://www.HGVS.org/varnomen).

## 3 RESULTS

**Figure 1:**
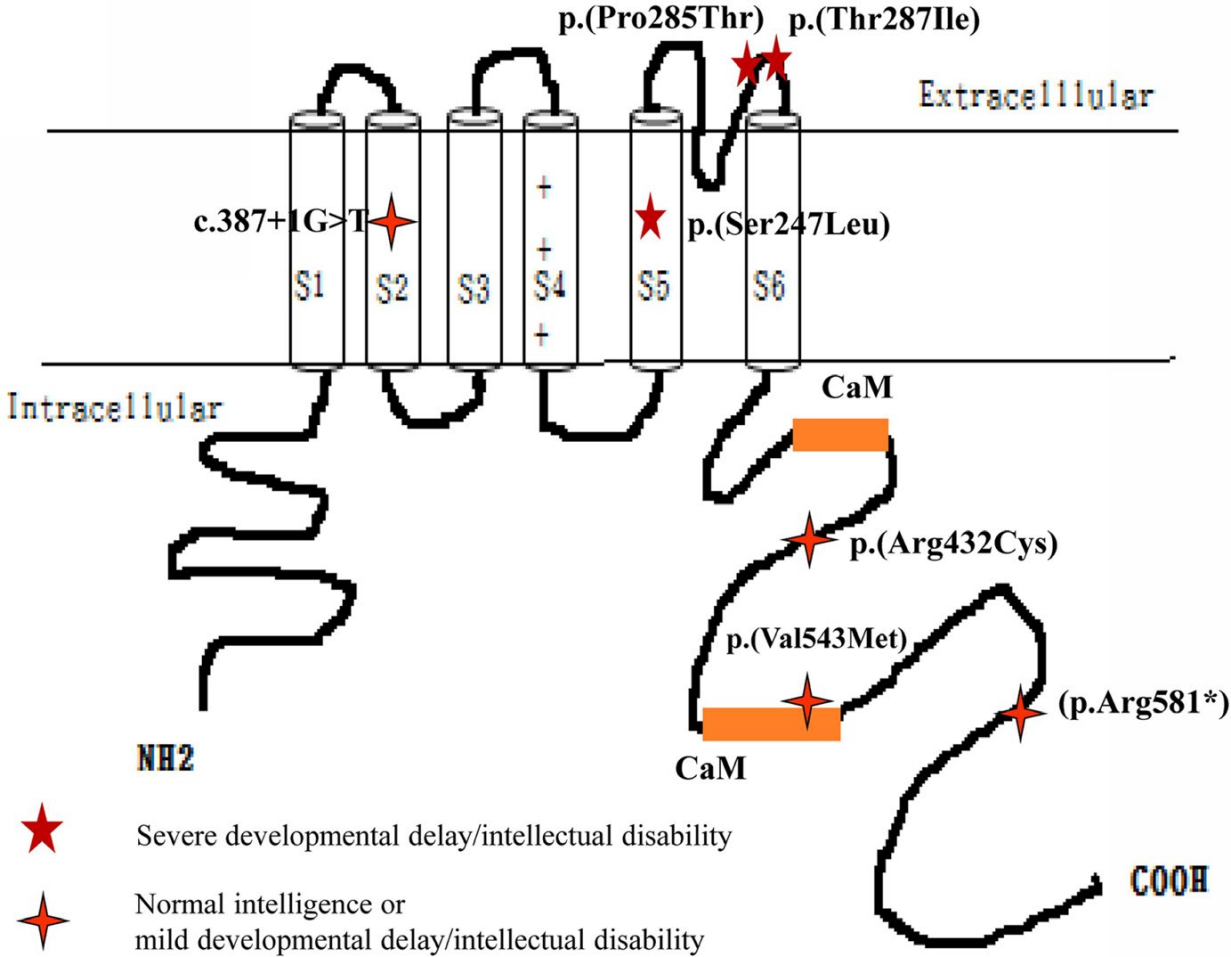
Seven mutation variants associated with *KCNQ2* functional domains. CaM: calmodulin domain

Seven (5%) of the 131 patients (three boys; four girls) had *KCNQ2* mutations: one each for c.1627 G>A c.1627 G>A p.(Val543Met); c.1294 C>T p.(Arg432Cys); c.740 C>T p.(Ser247Leu); c.1741 C>T (p.Arg581*); c.853 C>A p.(Pro285Thr); c.860 C>T p.(Thr287Ile); and a splicing mutation: c.387+1 G>T (Tables 1 and 2). None of these seven mutations was found in the control group (Supplemental Table S1). All seven mutations, except one splicing and one nonsense mutation, were missense. Four parents had the same mutations as did their children, but three children had de novo mutations: p.(Ser247Leu), p.(Pro285Thr), and p.(Thr287Ile). Mutation p.(Ser247Leu) was in the S5 transmembrane domain; mutations p.(Pro285Thr) and p.(Thr287Ile) were in the pore domain (Figure 1); the mutation in patient 3 was in a splicing site in the S2 domain (Figure 1); and mutation p.(Val543Met) was in the C‐terminal CaM domain (Figure 1).

**Table 1:** Seven of the 131 patients had identified *KCNQ2*mutatoins. The clinical and familial histories are summarized

Patient number	Patient 1	Patient 2	Patient 3	Patient 4	Patient 5	Patient 6	Patient 7
Genotype	c.1627 G>A	c.1294 C>T	c.387+1 G>T	c.740 C>T	c.1741 C>T	c.853 C>A	c.860 C>T
Protein change	p.(Val543Met)	p.(Arg432Cys)	Splicing	p.(Ser247Leu)	(p.Arg581*)	p.(Pro285Thr)	p.(Thr287Ile)
Family mutation	Father and two aunts	Father	Mother	De novo	Mother and sister	De novo	De novo
Sex	Female	Female	Male	Male	Female	Female	Male
Other genetic study	*KCNQ3*	Panel	*KCNQ3*	Whole exon	Panel	Whole exon	*KCNQ3*
Family number of seizures other than index patient (*n*)	3	1	1	0	2	0	0
Later seizures older than 3 years from familial *KCNQ2* mutation (*n*)	0	2	0	1	1	1	1
Age at first seizure	Day 14	1 year (febrile seizure)	Day 3	Day 3	Day 3	Day 2	Newborn
Seizure type	General tonic	General tonic	General clonic	General tonic	General tonic	General tonic	General tonic
Seizure frequency before drug control	+	+	+++	+++	+++	+++	+++
Drug control	OXC	OXC, TOP	PB, OXC	Intravenous PB, PHT then changed to PB, SAB, CLN	PB	Intravenous PB, PHT then changed to oral PB, SAB, CLN, OXC	TOP, OXC
Seizure frequency after 6‐months drugs	**‐**	**‐**	**‐**	**‐**	**‐**	**‐**	**+**
Abnormal MRI	No	No	No	Basal ganglion	No	Thin corpus callosum	No
Dev. Del./Int. Dis.	No	Mild(ADHD)	No	Severe	No	Severe	Severe

Note

The sequence data of each patient were checked against the GenBank reference sequence and version number of *KCNQ2* gene (NM_172107.3).

Abbreviations: NA, not available; VOUS, variance of unknown significance; PHT, phenytoin; OXC, oxcarbazepine; VPA, valproic acid; TOP, topiramate; PB, phenobarbital; KEP, levetiracetam; SAB, vigabatrin; CLN, clonazepam; MRI, magnetic resonance imaging; EEG, electroencephalography; +++, daily; ++, weekly; +, less than weekly; ADHD, attention deficit and hyperactivity; Dev. Del./Int. Dis., Developmental delay/intellectual disability.

**Table 2:** Genotypes and phenotypes in seven *KCNQ2* mutations

Genotype (*N*)		Phenotype	Severity	NCBI ClinVar	Functional domain	Global MAF	East Asia MAF	Cont. in Taiwan (*N* = 55)	FATHMM predict	Poly Phen2	SIFT	ACMG score
c.387+1 G>T	Splicing	BFNC	+	Pathogenic	S2	0	0	0				PVS1, PM2, PP4
c.740C>T p.(Ser247Leu)	Missense	EE	+++	Pathogenic	S5	0	0	0	D	D	D	PS2, PM1, PM2,PP3, PP4
c.853C>A p.(Pro285Thr)	Missense	EE	+++	Novel	Pore domain	0	0	0	D	D	D	PS2, PM1,PM2, PP3, PP4, PM5
c.860C>T p.(Thr287Ile)	Missense	EE	+++	VOUS	Pore domain	0	0	0	D	D	D	PS2, PM1, PM2, PP4, PP3
c.1294C>T p.(Arg432Cys)	Missense	CSWS	+	VOUS	C‐terminal	4.53E−05	0	0	D	D	D	PM2, PP3
c.1627G>A p.(Val543Met)	Missense	BFNC	+	VOUS	Calmodulin	0	0	0	D	D	D	PS3, PM2, PP3, PP4
c.1741 C>T (p.Arg581*)	Nonsense	BFNC	+	Pathogenic	C‐terminal	0	0	0				PVS1, PM2, PP4

Note

The sequence data of each patient were checked against the GenBank reference sequence and version number of *KCNQ2* gene (NM_172107.3).

Abbreviations: BFNC, benign familial neonatal convulsions; EE, neonatal‐onset epileptic encephalopathy; CSWS, continuous spikes and waves during slow‐wave sleep; NCBI ClinVar: National Center for Biotechnology Information, clinical variability and predictability (https://www.ncbi.nlm.nih.gov/clinvar); Cont, Control of healthy adults without seizures; Global MAF: global mutation allele frequency in EXAC browser; East Asia MAF: East Asia mutation allele frequency in EXAC browser; VOUS, variant of uncertain significance; FATHMM predict: Functional Analysis Through Hidden Markov Models prediction; D, damage; T, tolerant. ACMG, American College of Medical Genetics and Genomics and the Association for Molecular Pathology.

**Figure 2:**
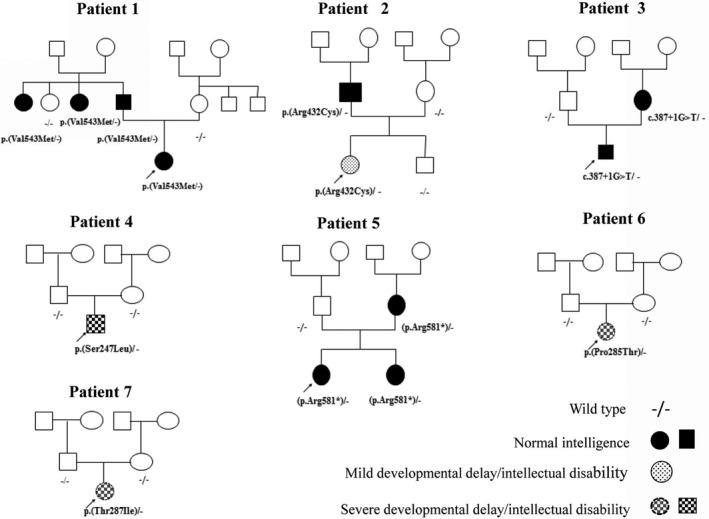
The pedigrees in seven index families are shown

The three patients with the de novo p.(Ser247Leu), p.(Pro285Thr), and p.(Thr287Ile) mutations had neonatal EE (Table 1). Three index patients had BFNC. One index patient with p.(Arg432Cys) had rolandic spikes in EEGs when awakening and prominent spikes when sleeping, which is consistent with continuous spikes and waves during slow‐wave sleep (CSWS) (Table 1). The pedigrees of the seven patients’ families are shown in Figure 2.

For patients whose first seizure onset occurred before they were 2 months old, the positive rate for a *KCNQ2* mutation was 13% (6/45), and the negative rate was 87% (39/45). This was significantly (*p* < 0.001) different from that of patients whose first seizure onset occurred after they were 2 months old: their negative rate for a *KCNQ2* mutation was 99%.

### 3.1 Patients with sporadic *KCNQ2* mutations had neonatal‐onset EE and severe neurodevelopmental outcomes

#### 3.1.1 Patient 4 had the de novo p.(Ser247Leu) mutation in the S5 domain

**Figure 3:**
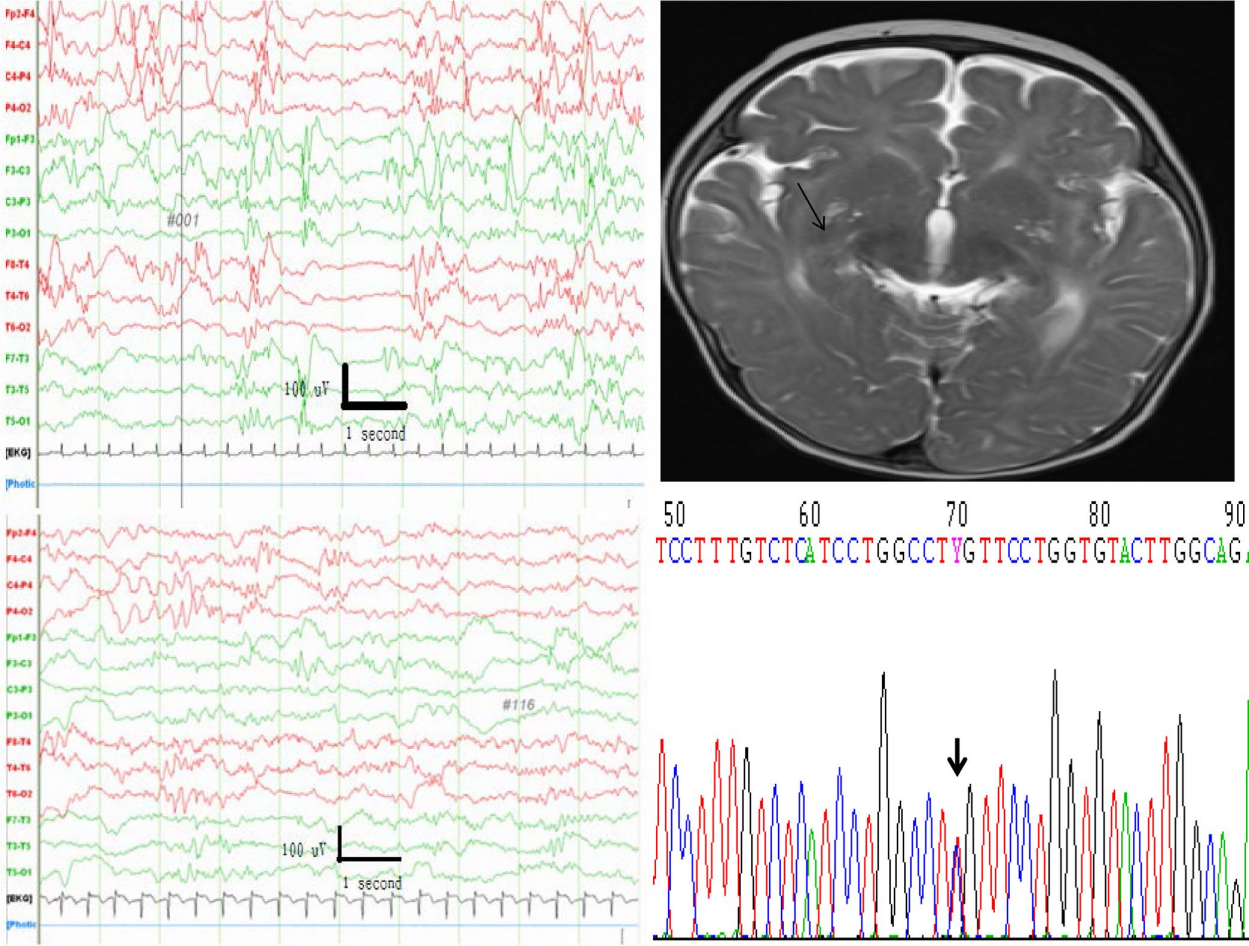
Sanger sequencing shows that patient 4 has a de novo c.740C>T p.(Ser247Leu) mutation (right lower, arrow), and an interictal EEG shows multiple focal spikes (left upper). The EEG improved after the patient turned 6 months old (left lower). His MRI shows hyperdensities (right upper, arrow) in his bilateral basal ganglia. He had frequent (>10 per day) neonatal seizures

Patient 4 had the c.740 C>T p.(Ser247Leu) mutation (Figure 3), frequent neonatal seizures, and apnea. His EEG shows multiple focal spikes (Figure 3). He was treated with multiple antiseizure drugs: phenobarbital, vigabatrin, and clonazepam. After he had turned 4 months old, he was treated with oxcarbazepine (OXC) and his seizures abated. An MRI showed basal ganglion hyperdensity (Figure 3). He also had a severe cognitive disability and could not walk at 3 years old.

#### 3.1.2 Patients 6 and 7 had the de novo p.(Pro285Thr) and p.(Thr287Ile) mutations in the pore domain

**Figure 4:**
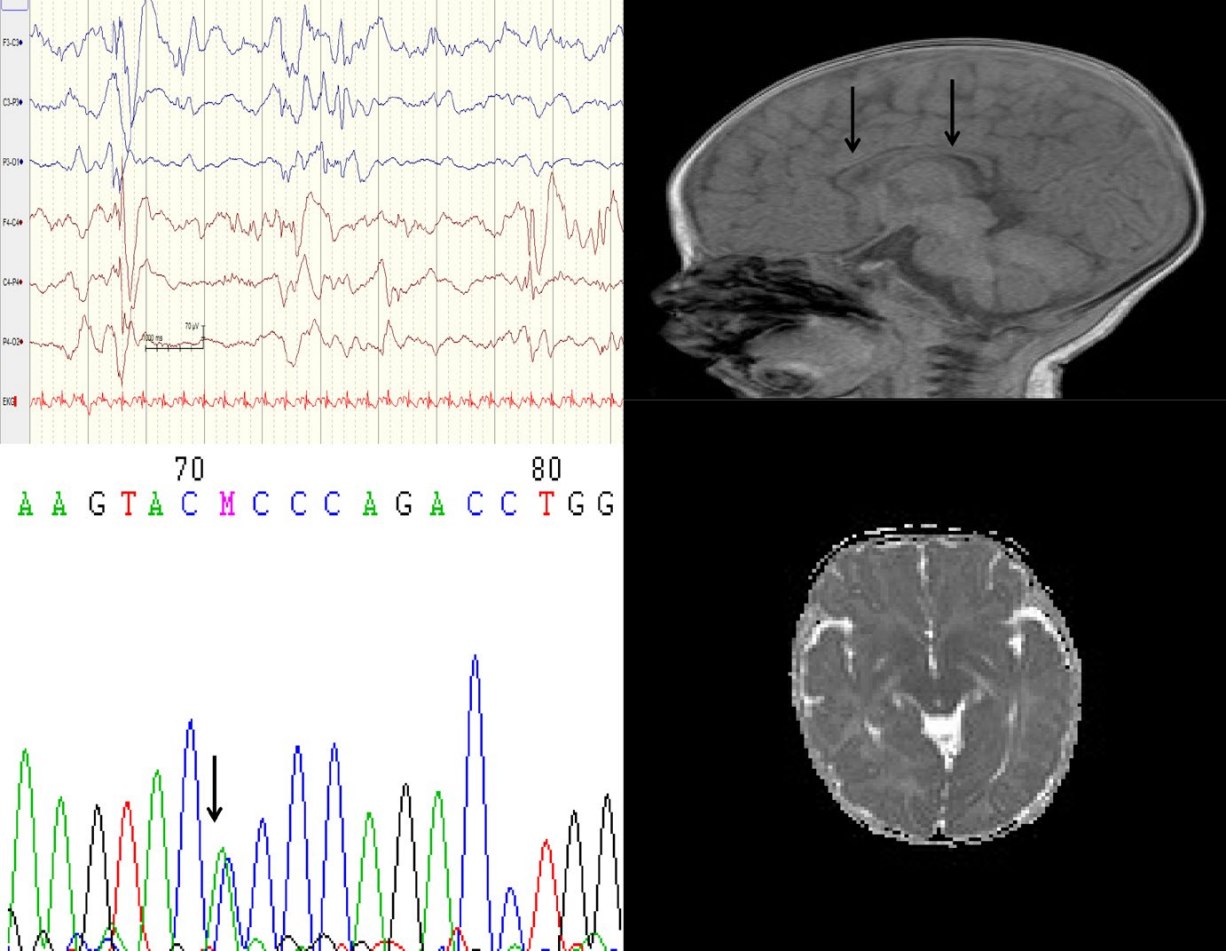
Patient 6 has a de novo c.853C>A p.(Pro285Thr) mutation (left lower) and an EEG that shows neonatal epileptic encephalopathy with burst‐suppression (left upper). Her EEG improved and her seizures attenuated after 2 months. The MRI shows a thin corpus callosum (right upper, arrows, and right lower)

**Figure 5:**
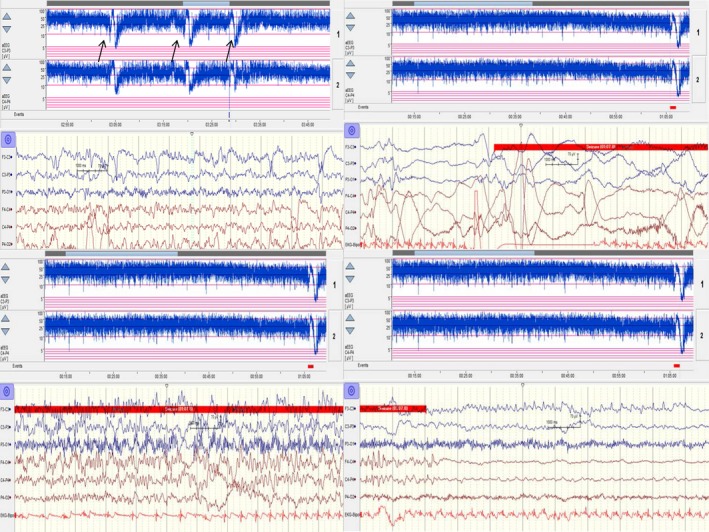
Patient 6’s amplitude‐integrated EEG monitor showed many seizures (arrows) with unique low‐voltage fast activity arising from the left hemisphere followed by rhythmic theta and delta rhythms and postictal extremely low‐voltage activity during ictal recordings

Patient 6 had the de novo p.(Pro285Thr) mutation, frequent neonatal seizures, and apnea. Her EEG (Figure 4) showed burst suppression. Her amplitude‐integrated EEG (*aEEG*) monitor showed many seizures (Figure 5: arrows) with unique low‐voltage fast activity arising from the left hemisphere and followed by rhythmic theta and delta rhythms and postictal extremely low‐voltage activity during ictal recordings. She had neonatal seizures and apnea. The seizures became less frequent after she turned 2 months old, but she had a severe cognitive disability. The patient was treated with multiple antiseizure drugs: phenobarbital, phenytoin, vigabatrin, and clonazepam. The seizures abated 2 months after she had been treated with OXC.

Patient 7 had the de novo p.(Thr287Ile) (Supplementary Figure S1: lower) and presented with neonatal seizures. OXC and topiramate controlled his seizures. He could walk, but he had a severe cognitive disability at 3 years old.

### 3.2 Patients with familial *KCNQ2* mutations had relatively benign neurodevelopmental outcomes

#### 3.2.1 Patient 2 had the c.1294 C>T p.(Arg432Cys) mutation

The computer‐based SIFT and PolyPhen algorithms predicted that the p.(Arg432Cys) mutation was deleterious. The Arginine (R) at protein position 432 is highly conserved in mammals. The patient had a mild epileptic phenotype; her first seizure (febrile) was at 1 year old, and she had an afebrile seizure at 5 years old. She was then treated with OXC, had two more seizures, and was referred to the hospital. After topiramate (2 mg kg^‐1^ day^‐1^) had been added, her seizures remitted. She had mild cognitive impairment. A genetic study showed that both she and her father, who had seizures as an infant, had the p.(Arg432Cys) mutation. An extensive epileptic panel study that included 203 genes found no other genetic defect responsible for her seizures (Supplementary Table S2). Her EEG showed bilateral central spikes during awakening and prominent spikes after sleeping. The spike waves clearly activated during sleep, compared with their EEG tracings while awake. The spike‐wave index is over 50% in non‐REM sleep and there were fewer sleep spindles visible, which is consistent with CSWS (Supplementary Figure S2) (Lesca et al., 2012; Scheltens‐de Boer, 2009). Her MRI was unremarkable, and her seizures remitted after she turned 7 years old.

#### 3.2.2 Patient 1 had the c.1627 G>A p.(Val543Met) mutation

This case was previously reported (Lee, Yang, Liang, et al., 2017). The patient's first seizure occurred when she was 2 weeks old. She had asymmetrical general tonic seizures. Her most recent previous seizure had been when she was 2 months old. Her seizures remitted after she had begun taking OXC when she was 2 months old. Her MRI was unremarkable. The family history showed that the patient's father and two of her aunts also had the p.(Val543Met) mutation and had had seizures from birth, but they had no cognitive disabilities. At 6 years and 2 months old, her cognitive development was normal.

#### 3.2.3 Patient 3 had the c.387+1 G>T (splicing) mutation

Patient 3’s first seizure occurred when he was 3 days old. The seizures were general tonic‐clonic seizures with lip cyanosis. He was first treated with intravenous phenobarbital, after which the seizures temporarily remitted. At 1 year old, the patient had another general tonic‐clonic seizure and was treated with OXC. His MRI and neurodevelopment were unremarkable. The family history showed that the patient's mother had had seizures from birth. A genetic study for *KCNQ2* showed that both he and his mother had the c.387+1 G>T mutation but no *KCNQ3* mutation (Supplementary Figure S1: upper).

#### 3.2.4 Patient 5 had the c.1741 C>T (p.Arg581*) mutation

Patient 5’s first seizure was a general tonic‐clonic seizure with lip cyanosis when she was 3 days old. The seizures remitted after she had been treated with oral phenobarbital. Her MRI and neurodevelopment were unremarkable at 4 years old. The family history showed that the patient's mother and sister had had seizures from birth. A genetic study for *KCNQ2* showed that all three had the c.1741 C>T(p.Arg581*) mutation.

### 3.3 Age at onset of seizures, and relapse of seizures after 3 years old

Seizure onset occurred in 6 of the 7 index patients when they were younger than 1 month and in one index patient when she was older than 1 month (febrile seizures at 1 year old). In the seven families, we found 14 patients with confirmed *KCNQ2* mutations. Seizure onset occurred at younger than 1 month in 12 (86%) and at older than 1 month in 2 (14%) (1 with infantile seizures (1 month to 1 year old); 1 with febrile seizures at 1 year old).

We confirmed *KCNQ2* mutations in 14 patients, six (43%) of whose seizures continued after they were older than 3 years. All seven index patients had general tonic or clonic seizures. EEGs showed burst suppression or multiple focal spikes in three index patients, focal discharges in 3, and CSWS in 1.

### 3.4 Drug treatment

One of the seven index patients was treated with only one antiseizure drug (patient 1: OXC); six were treated with more than 1 (four were treated with OXC, and their seizures remitted). Seizures in all seven patients completely or partially remitted after 6 months of drug treatments (Table 1).

## 4 DISCUSSION

Our most important finding is that *KCNQ2* mutations led to a variety of phenotypes in childhood epilepsy, for example, neonatal‐onset EE, BFNC, and CSWS. We found *KCNQ2* mutations in about 5% of nonlesional childhood epilepsy patients, and in about 13% of patients with neonatal seizure onset when they were younger than 2 months. We found three de novo mutations (p.(Ser247Leu), p.(Pro285Thr), and p.(Thr287Ile)), all of which are in critical *KCNQ2* domains. p.(Pro285Thr) is a novel mutation. Patients with one of these de novo mutations had worse outcomes.

The p.(Ser247Leu) and p.(Ser247Trp) mutations are pathogenic (https://www.ncbi.nlm.nih.gov/clinvar), as is (p.Ser247*) (Dedek, Fusco, Teloy, & Steinlein, 2003). Interestingly, each leads to a different phenotype. The p.(Ser247Leu) and p.(Ser247Trp) cause neonatal‐onset EE; however, (p.Ser247*) causes benign neonatal convulsions(Hunter et al., 2006). Kato et al. (2013) reported a de novo mutation p.(Pro285His**)** that causes Ohtahara syndrome. The global allele frequencies of p.(Ser247Leu), p.(Pro285Thr), and p.(Thr287Ile) are zero, according to the ExAC browser (http://exac.broadinstitute.org/). In our case, p.(Pro285Thr) was de novo, novel, and highly likely to cause neonatal‐onset EE, according to the guidelines of the American College of Medical Genetics and Genomics and the Association for Molecular Pathology (ACMG) (Richards et al., 2015). The p.(Thr287Ile) was classified as a variant of uncertain significance (VOUS) (https://www.ncbi.nlm.nih.gov/clinvar). In patient 7, who had the p.(Thr287Ile) variant, p.(Thr 287Asn) was also pathogenic (Milh et al., 2013), which supports the finding that p.(Thr287Ile) is pathogenic. We hypothesize that p.(Ser247Leu), p.(Pro285Thr), and p.(Thr287Ile) contribute to neonatal‐onset EE, and that all three should be classified as pathogenic.

We also found that *KCNQ2*‐associated epilepsy patients had varied outcomes. Two regions involved with the important *KCNQ2* functional domains—S1–S6 and CaM—might cause neonatal‐onset BFNC. However, in the most benign cases, the neurodevelopmental outcomes were relatively better. If the epilepsy is hereditary, seizure remission usually occurs after the patient turns 3 years old, and most such patients undergo normal cognitive development (Claes et al., 2004; Grinton et al., 2015; Singh et al., 2003).

We also found c.1545 G>C p.(Glu515Asp) in five patients with varied phenotypes: BFNC, CSWS, and unclassified epilepsy syndrome. However, they were excluded from the case series because of conflicting interpretations of p.(Glu515Asp)’s pathogenicity, despite our report (Lee, Yang, & Li, 2017) that there was a functional current change in HEK293 cells transfected with p.(Glu515Asp). Patients with p.(Glu515Asp) were associated with a mild BFNC phenotype, but in some patients, it was associated with cognitive delay and attention‐deficit hyperactive disorder (ADHD). Dravet syndrome patients with the p.(Glu515 Asp) mutation more often have developmental delays than do Dravet syndrome patients without p.(Glu515Asp) (Hammer et al., 2017). It is probable that other mutations of modified genes contributed to the phenotype. The global allele frequency of p.(Glu515Asp) is 0.002499, according to the ExAC browser (http://exac.broadinstitute.org/). The Exac population includes patients with Tourette's syndrome and schizophrenia. Amino acid changes from E (glutamic acid) to D (aspartic acid) can cause a thermophilic change of protein. The p.(Glu515Asp) is in the CaM domain, which means that its functional position is relatively important. However, to determine the pathogenicity of the p.(Glu515Asp) mutation, additional accurate age‐matched case‐control studies are necessary. We also found three other benign *KCNQ2* variants: c.2264A>G p.(Tyr755Cys), c.1253G>T p.(Gly418Val), and c.51G>C p.(Glu17Asp). The p.(Glu17Asp) variant is novel, and after a segregation study, we hypothesized that it was benign.

Although BFNC are considered benign, BFNC patients might have cluster seizures, which inevitably require drug control to prevent secondary brain injury (Grinton et al., 2015). In neonatal EE patients, OXC, valproic acid, topiramate, vigabatrin, and clonazepam were used to treat seizures (Kato et al., 2013; Weckhuysen et al., 2013, 2012). OXC was considered more efficacious for *KCNQ2*‐associated seizures in several studies (Grinton et al., 2015; Pisano et al., 2015; Sands et al., 2016), which is consistent with our findings. However, responses to antiseizure drugs require additional investigation.

## 5 CONCLUSIONS

*KCNQ2* mutations accounted for 5% of all nonlesional pediatric epilepsy and 13% of patients with seizure onset before 2 months old. *KCNQ2* mutations can cause variable phenotypes in children, from BFNC to severe neonatal‐onset EE. The p.(Ser247Leu), p.(Pro 285Thr), and p.(Thr287Ile) mutations can cause neonatal‐onset EE.

## CONFLICT OF INTEREST

The authors declared that they have no conflict of interest.

## References

[mgg3816-bib-0001] Ambrosino, P. , Alaimo, A. , Bartollino, S. , Manocchio, L. , De Maria, M. , Mosca, I. , … Soldovieri, M. V. (2015). Epilepsy‐causing mutations in Kv7.2 C‐terminus affect binding and functional modulation by calmodulin. Biochimica Et Biophysica Acta, 1852(9), 1856–1866. 10.1016/j.bbadis.2015.06.012 26073431

[mgg3816-bib-0002] Biervert, C. , Schroeder, B. C. , Kubisch, C. , Berkovic, S. F. , Propping, P. , Jentsch, T. J. , & Steinlein, O. K. (1998). A potassium channel mutation in neonatal human epilepsy. Science, 279(5349), 403–406. 9430594 10.1126/science.279.5349.403

[mgg3816-bib-0003] Chen, D. Y. , Chowdhury, S. , Farnaes, L. , Friedman, J. R. , Honold, J. , Dimmock, D. P. , & Gold, O. (2018). Rapid diagnosis of KCNQ2‐associated early infantile epileptic encephalopathy improved outcome. Pediatric Neurology, 86, 69–70. 10.1016/j.pediatrneurol.2018.06.002 30107960 PMC6824418

[mgg3816-bib-0004] Choi, S. J. , Mukai, J. , Kvajo, M. , Xu, B. , Diamantopoulou, A. , Pitychoutis, P. M. , … Zhang, H. (2018). A schizophrenia‐related deletion leads to KCNQ2‐dependent abnormal dopaminergic modulation of prefrontal cortical interneuron activity. Cerebral Cortex, 28(6), 2175–2191. 10.1093/cercor/bhx123 28525574 PMC6018968

[mgg3816-bib-0005] Claes, L. R. , Ceulemans, B. , Audenaert, D. , Deprez, L. , Jansen, A. , Hasaerts, D. , … De Jonghe, P. (2004). De novo KCNQ2 mutations in patients with benign neonatal seizures. Neurology, 63(11), 2155–2158. 10.1212/01.WNL.0000145629.94338.89 15596769

[mgg3816-bib-0006] Cooper, E. C. , Harrington, E. , Jan, Y. N. , & Jan, L. Y. (2001). M channel KCNQ2 subunits are localized to key sites for control of neuronal network oscillations and synchronization in mouse brain. Journal of Neuroscience, 21(24), 9529–9540. 10.1523/JNEUROSCI.21-24-09529.2001 11739564 PMC6763050

[mgg3816-bib-0007] Cooper, E. C. , & Jan, L. Y. (2003). M‐channels: Neurological diseases, neuromodulation, and drug development. Archives of Neurology, 60(4), 496–500. 10.1001/archneur.60.4.496 12707061

[mgg3816-bib-0008] Coppola, G. , Castaldo, P. , Miraglia del Giudice, E. , Bellini, G. , Galasso, F. , Soldovieri, M. V. , … Taglialatela, M. (2003). A novel KCNQ2 K+ channel mutation in benign neonatal convulsions and centrotemporal spikes. Neurology, 61(1), 131–134. 12847176 10.1212/01.wnl.0000069465.53698.bd

[mgg3816-bib-0009] Dedek, K. , Fusco, L. , Teloy, N. , & Steinlein, O. K. (2003). Neonatal convulsions and epileptic encephalopathy in an Italian family with a missense mutation in the fifth transmembrane region of KCNQ2. Epilepsy Research, 54(1), 21–27. 10.1016/S0920-1211(03)00037-8 12742592

[mgg3816-bib-0010] Grinton, B. E. , Heron, S. E. , Pelekanos, J. T. , Zuberi, S. M. , Kivity, S. , Afawi, Z. , … Berkovic, S. F. (2015). Familial neonatal seizures in 36 families: Clinical and genetic features correlate with outcome. Epilepsia, 56(7), 1071–1080. 10.1111/epi.13020 25982755

[mgg3816-bib-0011] Hammer, M. F. , Ishii, A. , Johnstone, L. , Tchourbanov, A. , Lau, B. , Sprissler, R. , … Hirose, S. (2017). Rare variants of small effect size in neuronal excitability genes influence clinical outcome in Japanese cases of SCN1A truncation‐positive Dravet syndrome. PLoS ONE, 12(7), e0180485 10.1371/journal.pone.0180485 28686619 PMC5501540

[mgg3816-bib-0012] Hunter, J. , Maljevic, S. , Shankar, A. , Siegel, A. , Weissman, B. , Holt, P. , … Escayg, A. (2006). Subthreshold changes of voltage‐dependent activation of the K(V)7.2 channel in neonatal epilepsy. Neurobiology of Diseases, 24(1), 194–201. 10.1016/j.nbd.2006.06.011 16916607

[mgg3816-bib-0013] Kato, M. , Yamagata, T. , Kubota, M. , Arai, H. , Yamashita, S. , Nakagawa, T. , … Saitsu, H. (2013). Clinical spectrum of early onset epileptic encephalopathies caused by KCNQ2 mutation. Epilepsia, 54(7), 1282–1287. 10.1111/epi.12200 23621294

[mgg3816-bib-0014] Lee, I. C. , Yang, J. J. , & Li, S. Y. (2017). A KCNQ2 E515D mutation associated with benign familial neonatal seizures and continuous spike and waves during slow‐wave sleep syndrome in Taiwan. Journal of the Formosan Medical Association, 116(9), 711–719. 10.1016/j.jfma.2016.11.009 28038823

[mgg3816-bib-0015] Lee, I. C. , Yang, J. J. , Liang, J. S. , Chang, T. M. , & Li, S. Y. (2017). KCNQ2‐associated neonatal epilepsy: Phenotype might correlate with genotype. Journal of Child Neurology, 32(8), 704–711. 10.1177/0883073817701873 28399683

[mgg3816-bib-0016] Lee, Y. H. , Kim, J. H. , & Song, G. G. (2013). Pathway analysis of a genome‐wide association study in schizophrenia. Gene, 525(1), 107–115. 10.1016/j.gene.2013.04.014 23644028

[mgg3816-bib-0017] Leppert, M. , Anderson, V. E. , Quattlebaum, T. , Stauffer, D. , O'Connell, P. , Nakamura, Y. , … White, R. (1989). Benign familial neonatal convulsions linked to genetic markers on chromosome 20. Nature, 337(6208), 647–648. 10.1038/337647a0 2918897

[mgg3816-bib-0018] Lerche, H. , Biervert, C. , Alekov, A. K. , Schleithoff, L. , Lindner, M. , Klingler, W. , … Steinlein, O. K. (1999). A reduced K+ current due to a novel mutation in KCNQ2 causes neonatal convulsions. Annals of Neurology, 46(3), 305–312. 10.1002/1531-8249(199909)46:3<305:AID-ANA5>3.0.CO;2-5 10482260

[mgg3816-bib-0019] Lesca, G. , Rudolf, G. , Labalme, A. , Hirsch, E. , Arzimanoglou, A. , Genton, P. , … Szepetowski, P. (2012). Epileptic encephalopathies of the Landau‐Kleffner and continuous spike and waves during slow‐wave sleep types: Genomic dissection makes the link with autism. Epilepsia, 53(9), 1526–1538. 10.1111/j.1528-1167.2012.03559.x 22738016

[mgg3816-bib-0020] Lindy, A. S. , Stosser, M. B. , Butler, E. , Downtain‐Pickersgill, C. , Shanmugham, A. , Retterer, K. , … McKnight, D. A. (2018). Diagnostic outcomes for genetic testing of 70 genes in 8565 patients with epilepsy and neurodevelopmental disorders. Epilepsia, 59(5), 1062–1071. 10.1111/epi.14074 29655203

[mgg3816-bib-0021] Liu, W. , & Devaux, J. J. (2014). Calmodulin orchestrates the heteromeric assembly and the trafficking of KCNQ2/3 (Kv7.2/3) channels in neurons. Molecular and Cellular Neurosciences, 58, 40–52. 10.1016/j.mcn.2013.12.005 24333508

[mgg3816-bib-0022] Maljevic, S. , Naros, G. , Yalcin, O. , Blazevic, D. , Loeffler, H. , Caglayan, H. , … Lerche, H. (2011). Temperature and pharmacological rescue of a folding‐defective, dominant‐negative KV 7.2 mutation associated with neonatal seizures. Human Mutation, 32(10), E2283–2293. 10.1002/humu.21554 21913284

[mgg3816-bib-0023] Maljevic, S. , Wuttke, T. V. , & Lerche, H. (2008). Nervous system KV7 disorders: Breakdown of a subthreshold brake. Journal of Physiology, 586(7), 1791–1801. 10.1113/jphysiol.2008.150656 18238816 PMC2375730

[mgg3816-bib-0024] Miceli, F. , Soldovieri, M. V. , Ambrosino, P. , De Maria, M. , Migliore, M. , Migliore, R. , & Taglialatela, M. (2015). Early‐onset epileptic encephalopathy caused by gain‐of‐function mutations in the voltage sensor of Kv7.2 and Kv7.3 potassium channel subunits. Journal of Neuroscience, 35(9), 3782–3793. 10.1523/jneurosci.4423-14.2015 25740509 PMC6605567

[mgg3816-bib-0025] Milh, M. , Boutry‐Kryza, N. , Sutera‐Sardo, J. , Mignot, C. , Auvin, S. , Lacoste, C. , … Villard, L. (2013). Similar early characteristics but variable neurological outcome of patients with a de novo mutation of KCNQ2. Orphanet Journal of Rare Diseases, 8, 80 10.1186/1750-1172-8-80 23692823 PMC3670812

[mgg3816-bib-0026] Millichap, J. J. , Miceli, F. , De Maria, M. , Keator, C. , Joshi, N. , Tran, B. , … Taglialatela, M. (2017). Infantile spasms and encephalopathy without preceding neonatal seizures caused by KCNQ2 R198Q, a gain‐of‐function variant. Epilepsia, 58(1), e10–e15. 10.1111/epi.13601 27861786 PMC5219941

[mgg3816-bib-0027] Neubauer, B. A. , Waldegger, S. , Heinzinger, J. , Hahn, A. , Kurlemann, G. , Fiedler, B. , … Sander, T. (2008). KCNQ2 and KCNQ3 mutations contribute to different idiopathic epilepsy syndromes. Neurology, 71(3), 177–183. 10.1212/01.wnl.0000317090.92185.ec 18625963

[mgg3816-bib-0028] Pisano, T. , Numis, A. L. , Heavin, S. B. , Weckhuysen, S. , Angriman, M. , Suls, A. , … Cilio, M. R. (2015). Early and effective treatment of KCNQ2 encephalopathy. Epilepsia, 56(5), 685–691. 10.1111/epi.12984 25880994

[mgg3816-bib-0029] Richards, S. , Aziz, N. , Bale, S. , Bick, D. , Das, S. , Gastier‐Foster, J. , … Rehm, H. L. (2015). Standards and guidelines for the interpretation of sequence variants: A joint consensus recommendation of the American College of Medical Genetics and Genomics and the Association for Molecular Pathology. Genetics in Medicine, 17(5), 405–424. 10.1038/gim.2015.30 25741868 PMC4544753

[mgg3816-bib-0030] Sands, T. T. , Balestri, M. , Bellini, G. , Mulkey, S. B. , Danhaive, O. , Bakken, E. H. , … Cilio, M. R. (2016). Rapid and safe response to low‐dose carbamazepine in neonatal epilepsy. Epilepsia, 57(12), 2019–2030. 10.1111/epi.13596 27888506

[mgg3816-bib-0031] Scheltens‐de Boer, M. (2009). Guidelines for EEG in encephalopathy related to ESES/CSWS in children. Epilepsia, 50(Suppl 7), 13–17. 10.1111/j.1528-1167.2009.02211.x 19682043

[mgg3816-bib-0032] Singh, N. A. , Charlier, C. , Stauffer, D. , DuPont, B. R. , Leach, R. J. , Melis, R. , … Leppert, M. (1998). A novel potassium channel gene, KCNQ2, is mutated in an inherited epilepsy of newborns. Nature Genetics, 18(1), 25–29. 10.1038/ng0198-25 9425895

[mgg3816-bib-0033] Singh, N. A. , Westenskow, P. , Charlier, C. , Pappas, C. , Leslie, J. , Dillon, J. , … Leppert, M. F. (2003). KCNQ2 and KCNQ3 potassium channel genes in benign familial neonatal convulsions: Expansion of the functional and mutation spectrum. Brain, 126(Pt 12), 2726–2737. 10.1093/brain/awg286 14534157

[mgg3816-bib-0034] Wang, H. S. , Pan, Z. , Shi, W. , Brown, B. S. , Wymore, R. S. , Cohen, I. S. , … McKinnon, D. (1998). KCNQ2 and KCNQ3 potassium channel subunits: Molecular correlates of the M‐channel. Science, 282(5395), 1890–1893. 9836639 10.1126/science.282.5395.1890

[mgg3816-bib-0035] Weckhuysen, S. , Ivanovic, V. , Hendrickx, R. , Van Coster, R. , Hjalgrim, H. , Moller, R. S. , … De Jonghe, P. (2013). Extending the KCNQ2 encephalopathy spectrum: Clinical and neuroimaging findings in 17 patients. Neurology, 81(19), 1697–1703. 10.1212/01.wnl.0000435296.72400.a1 24107868 PMC3812107

[mgg3816-bib-0036] Weckhuysen, S. , Mandelstam, S. , Suls, A. , Audenaert, D. , Deconinck, T. , Claes, L. R. F. , … de Jonghe, P. (2012). KCNQ2 encephalopathy: Emerging phenotype of a neonatal epileptic encephalopathy. Annals of Neurology, 71(1), 15–25. 10.1002/ana.22644 22275249

[mgg3816-bib-0037] Wuttke, T. V. , Penzien, J. , Fauler, M. , Seebohm, G. , Lehmann‐Horn, F. , Lerche, H. , & Jurkat‐Rott, K. (2008). Neutralization of a negative charge in the S1–S2 region of the KV7.2 (KCNQ2) channel affects voltage‐dependent activation in neonatal epilepsy. Journal of Physiology, 586(2), 545–555. 10.1113/jphysiol.2007.143826 18006581 PMC2375582

[mgg3816-bib-0038] Zhou, X. , Zhuang, F. , Li, H. , Zheng, K. , Hong, Z. , Feng, W. , … Chen, J. (2016). Calmodulin regulates KCNQ2 function in epilepsy. American Journal of Translational Research, 8(12), 5610–5618. 28078031 PMC5209511

