# Supplementary figures and images for: KCNQ2 mutations in childhood nonlesional epilepsy: Variable phenotypes and a novel mutation in a case series
Source: Mol Genet Genomic Med. 2019 Jun 14;7(7):e00816. doi: 10.1002/mgg3.816 (PMC6625149; doi:10.1002/mgg3.816)

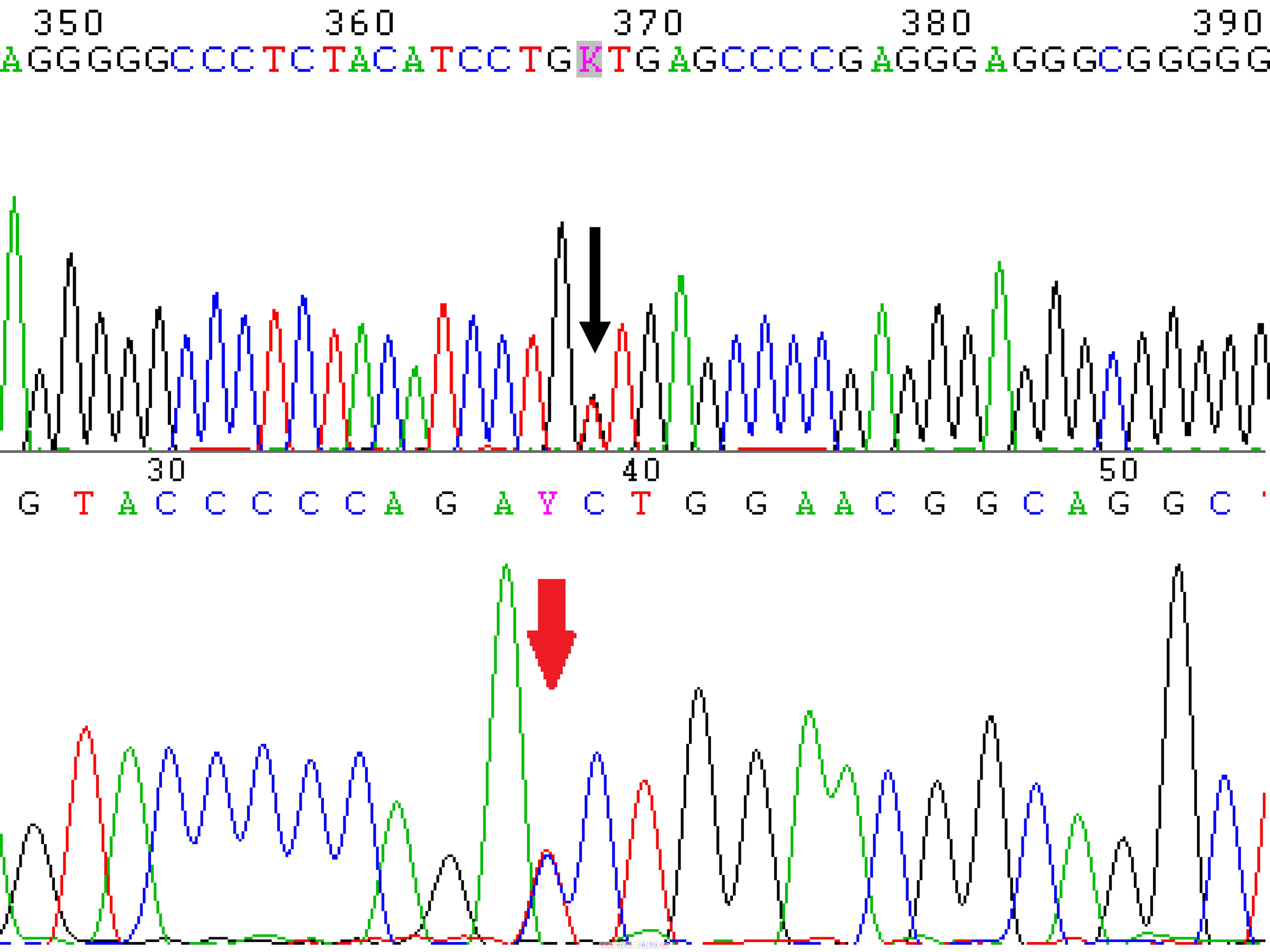

Supplement: Supplementary file 1 [file MGG3-7-e00816-s001.tif]

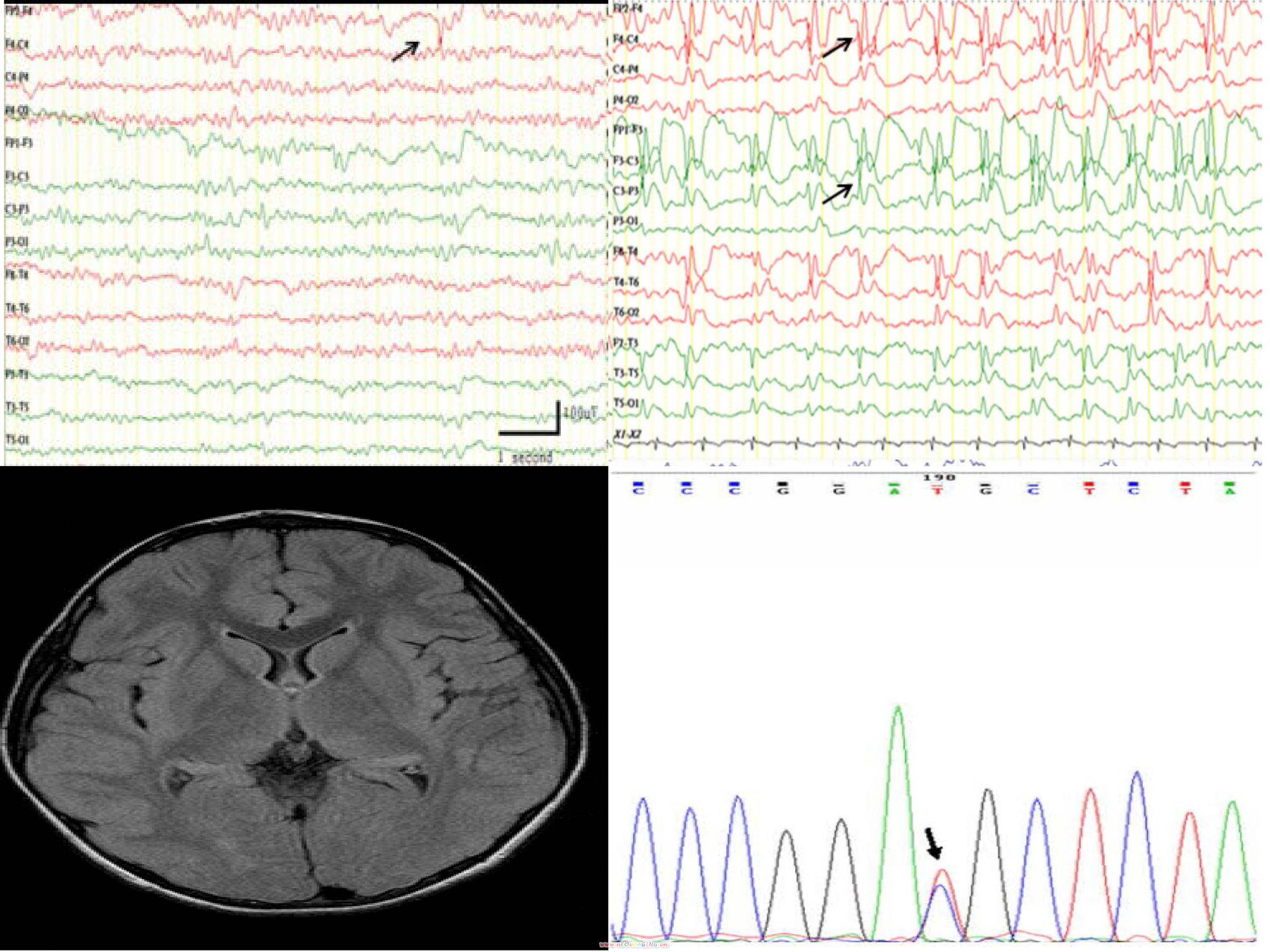

Supplement: Supplementary file 2 [file MGG3-7-e00816-s002.tif]
