# Supplementary material for: KCNQ2 mutations in childhood nonlesional epilepsy: Variable phenotypes and a novel mutation in a case series
Source: Mol Genet Genomic Med. 2019 Jun 14;7(7):e00816. doi: 10.1002/mgg3.816 (PMC6625149; doi:10.1002/mgg3.816)
Supplement: Supplementary file 3 [file MGG3-7-e00816-s003.doc]

Supplementary Table 1. Genotypes from 55 adults without a history of seizures.

|  |  | Chr | Position | Type | Zygosity | Genotype | Ref | Var | Var Freq  (%) | dbSNP | AA Var |
| --- | --- | --- | --- | --- | --- | --- | --- | --- | --- | --- | --- |
| Econ 17 | c.2619+248 C > T | chr20 | 62037749 | SNP | Het | G/A | G | A | 41.8 | rs6122440 |  |
| Exon 17 | c.2238 T > A | chr20 | 62038378 | SNP | Het | A/T | A | T | 14.5 | rs1801471 | p.(Pro746=) |
| Intron 16 | c.1888-29G > A | chr20 | 62038757 | SNP | Het | C/T | C | T | 18.2 | rs3746364 |  |
| Exon 15 | c.1719C > T | chr20 | 62044847 | SNP | Het | G/A | G | A | 3.6 | N/A | p.(Ala573=) |
| Intron 13 | c.1525+57C > T | chr20 | 62046199 | SNP | Het | G/A | G | A | 9.1 | N/A |  |
| Intron 13 | c.1525+55G > A | chr20 | 62046201 | SNP | Het | C/T | C | T | 7.3 | N/A |  |
| Intron 13 | c.1525+53T > C | chr20 | 62046203 | SNP | Het | A/G | A | G | 12.7 | N/A |  |
| Exon 6 | c.912C > T | chr20 | 62070966 | SNP | Het | G/A | G | A | 40.0 | rs2297385 | p.(Phe304=) |
| Intron 2 | c.388-26G > T | chr20 | 62076743 | SNP | Het | C/A | C | A | 6.7 | rs6062939 |  |
| Intron 1 | c.296+112T > A | chr20 | 62103409 | SNP | Het | A/T | A | T | 27.3 | N/A |  |
| Exon 1 | c.127G > A | chr20 | 62103690 | SNP | Het | C/T | C | T | 25.4 | N/A | p.(Ala43Thr) |

Chr,chromosome; het, SNP, single-nucleotide polymorphism; heterozygous; ref, reference; var, variance; fre, frequency; AA Var, amino acid variance; N/A, non available. The sequence data of each patient were checked against the GenBank reference sequence and version number of *KCNQ2* gene (NM_172107.**3**).
