# Supplementary material for: KCNQ2 mutations in childhood nonlesional epilepsy: Variable phenotypes and a novel mutation in a case series
Source: Mol Genet Genomic Med. 2019 Jun 14;7(7):e00816. doi: 10.1002/mgg3.816 (PMC6625149; doi:10.1002/mgg3.816)
Supplement: Supplementary file 4 [file MGG3-7-e00816-s004.docx]

| *AARS* (16q22.1) | [*CLN6* (15q21-q23)](http://www.ncbi.nlm.nih.gov/gtr/genes/54982/) | [*HCN4* (15q24-q25)](http://www.ncbi.nlm.nih.gov/gtr/genes/10021/) | | [*NRXN1* (2p16.3)](http://www.ncbi.nlm.nih.gov/gtr/genes/9378/) | *SLC12A5* (20q13.12) |
| --- | --- | --- | --- | --- | --- |
| [*ABAT* (16p13.3)](http://www.ncbi.nlm.nih.gov/gtr/genes/18/) | [*CLN8* (8p23)](http://www.ncbi.nlm.nih.gov/gtr/genes/2055/) | *HDAC2* (6q21) | | *NTNG1* (1p13.3) | *SLC13A5* (17p13.1) |
| *ABCD1* (Xq28) | [*CNTNAP2* (7q35-q36)](http://www.ncbi.nlm.nih.gov/gtr/genes/26047/) | *HDAC4* (2q37.3) | | [*OPHN1* (Xq12)](http://www.ncbi.nlm.nih.gov/gtr/genes/4983/) | *SLC1A1* (9p24.2) |
| [*ABCB1* (7q21.1)](http://www.ncbi.nlm.nih.gov/gtr/genes/5243/) | *CPA6* (8q13.2) | *HNRNPU* (1q44) | | [*OPRM1* (6q24-q25)](http://www.ncbi.nlm.nih.gov/gtr/genes/4988/) | *SLC25A12* (2q31.1) |
| *ADGRV1*(5q14.3) | *CSNK1G1* (15q22.31) | *HUWE1* (Xp11.22) | | [*PAFAH1B1* (17p13.3)](http://www.ncbi.nlm.nih.gov/gtr/genes/5048/) | [*SLC25A19* (17q25.3)](http://www.ncbi.nlm.nih.gov/gtr/genes/60386/) |
| [*ADSL* (22q13.1)](http://www.ncbi.nlm.nih.gov/gtr/genes/158/) | [*CSTB* (21q22.3)](http://www.ncbi.nlm.nih.gov/gtr/genes/1476/) | [*IER3IP1*](http://www.cureepilepsy.org/egi/genes/ier3ip1.asp) (18q21.1) | | [*PCDH19* (Xq22)](http://www.ncbi.nlm.nih.gov/gtr/genes/57526/) | [*SLC25A22* (11p15.5)](http://www.ncbi.nlm.nih.gov/gtr/genes/79751/) |
| [*ALG13*](http://www.cureepilepsy.org/egi/genes/alg13.asp) (Xq23) | [*CTSD* (11p15.5)](http://www.ncbi.nlm.nih.gov/gtr/genes/1509/) | *ITPA* (20p13) | | [*PCNT* (21q22.3)](http://www.ncbi.nlm.nih.gov/gtr/genes/5116/) | [*SLC35A2*](http://www.cureepilepsy.org/egi/genes/slc35a2.asp) (Xp11.23) |
| [*ALDH7A1* (5q31)](http://www.ncbi.nlm.nih.gov/gtr/genes/501/) | [*DCX* (Xq22.3-q23)](http://www.ncbi.nlm.nih.gov/gtr/genes/1641/) | [*KCNA1* (12p13)](http://www.ncbi.nlm.nih.gov/gtr/genes/3736/) | | *PDHA1* (Xp22.12) | *SLC6A1* (3P25.3) |
| [*ARFGEF2* (20q13.13)](http://www.ncbi.nlm.nih.gov/gtr/genes/10564/) | [*DEPDC5*](http://www.cureepilepsy.org/egi/genes/depdc5.asp) (22q12.2-q12.3) | [*KCNA2*](http://www.cureepilepsy.org/egi/genes/kcna2.asp) (1p13.3) | | [*PHF6* (Xq26.3)](http://www.ncbi.nlm.nih.gov/gtr/genes/84295/) | [*SLC2A1 (*1p35-p31.3)](http://www.ncbi.nlm.nih.gov/gtr/genes/6513/) |
| [*ARHGEF9* (Xq22.1)](http://www.ncbi.nlm.nih.gov/gtr/genes/23229/) | [*DNM1*](http://www.cureepilepsy.org/egi/genes/dnm1.asp)(9q34.11) | [*KCNB1*](http://www.cureepilepsy.org/egi/genes/kcnb1.asp) (20q13.13) | | *PIGA* (Xp22.2) | [*SLC9A6* (Xq26.3)](http://www.ncbi.nlm.nih.gov/gtr/genes/10479/) |
| *ARHGEF15*(17p13.1) | *DOCK7* (1p31.3) | [*KCNAB1* (3q26.1)](http://www.ncbi.nlm.nih.gov/gtr/genes/7881/) | | *PIGO* (9p13.3) | [*SNIP1*](http://www.cureepilepsy.org/egi/genes/snip1.asp) (1p34.3) |
| [*ARX* (Xp22.13)](http://www.ncbi.nlm.nih.gov/gtr/genes/170302/) | [*EFHC1* (6p12-p11)](http://www.ncbi.nlm.nih.gov/gtr/genes/114327/) | *KCNC1* (11p15.1) | | [*PLCB1* (20p12)](http://www.ncbi.nlm.nih.gov/gtr/genes/23236/) | *SMS* (11p11.2) |
| *ARV1*(1q42.2) | [*EEF1A2*](http://www.cureepilepsy.org/egi/genes/eef1a2.asp) (20q13.33) | *KCNH5* (14q23.2) | | [*PNKP* (19q13.4)](http://www.ncbi.nlm.nih.gov/gtr/genes/11284/) | [*SPTAN1* (9q33-q34)](http://www.ncbi.nlm.nih.gov/gtr/genes/6709/) |
| *ASAH1*(8p22) | [*EFHC2* (Xp11.3)](http://www.ncbi.nlm.nih.gov/gtr/genes/80258/) | [*KCNJ10* (1q23.2)](http://www.ncbi.nlm.nih.gov/gtr/genes/3766/) |  | [*PNPO* (17q21.32)](http://www.ncbi.nlm.nih.gov/gtr/genes/55163/) | [*SRPX2* (Xq21.33-q23)](http://www.ncbi.nlm.nih.gov/gtr/genes/27286/) |
| [*ASPM* (1q31)](http://www.ncbi.nlm.nih.gov/gtr/genes/259266/) | [*EMX2* (10q26.1)](http://www.ncbi.nlm.nih.gov/gtr/genes/2018/) | [*KCNJ11* (11p15.1)](http://www.ncbi.nlm.nih.gov/gtr/genes/3767/) |  | [*POLG* (15q25)](http://www.ncbi.nlm.nih.gov/gtr/genes/5428/) | [*STIL* (1p33)](http://www.ncbi.nlm.nih.gov/gtr/genes/6491/) |
| [*ATP1A2* (1q23.2)](http://www.ncbi.nlm.nih.gov/gtr/genes/477/) | [*EPM2A* (6q24)](http://www.ncbi.nlm.nih.gov/gtr/genes/7957/) | [*KCNMA1* (10q22.3)](http://www.ncbi.nlm.nih.gov/gtr/genes/3778/) |  | [*PPT1* (1p32)](http://www.ncbi.nlm.nih.gov/gtr/genes/5538/) | [*ST3GAL3*](http://www.cureepilepsy.org/egi/genes/st3gal3.asp) (1p34.1) |
| *ATP1A3* (19q13.2) | *FASN* (17q25.3) | [*KCNQ2* (20q13.3)](http://www.ncbi.nlm.nih.gov/gtr/genes/3785/) |  | [*PRICKLE1* (12q12)](http://www.ncbi.nlm.nih.gov/gtr/genes/144165/) | *ST3GAL5* (2p11.2) |
| [*ATP6AP2* (Xp11.4)](http://www.ncbi.nlm.nih.gov/gtr/genes/10159/) | [*FLNA* (Xq28)](http://www.ncbi.nlm.nih.gov/gtr/genes/2316/) | [*KCNQ3* (8q24)](http://www.ncbi.nlm.nih.gov/gtr/genes/3786/) |  | [*PRICKLE2* (3p14)](http://www.ncbi.nlm.nih.gov/gtr/genes/166336/) | [*STRADA*](http://www.cureepilepsy.org/egi/genes/strada.asp) (17q23.3) |
| *ATP7A* (Xq21.1) | [*FLVCR2* (14q24.3)](http://www.ncbi.nlm.nih.gov/gtr/genes/55640/) | [*KCNT1*](http://www.cureepilepsy.org/egi/genes/kcnt1.asp) (9q34.3) |  | [*PRRT2* (16p11.2)](http://www.ncbi.nlm.nih.gov/gtr/genes/112476/) | [*STXBP1* (9q34.1)](http://www.ncbi.nlm.nih.gov/gtr/genes/6812/) |
| [*AMT* (3p21.2-p21.1)](http://www.ncbi.nlm.nih.gov/gtr/genes/275/) | [*FOLR1* (11q13.3-q13.5)](http://www.ncbi.nlm.nih.gov/gtr/genes/2348/) | [*KCNV2* (9p24.2)](http://www.ncbi.nlm.nih.gov/gtr/genes/169522/) |  | [*PTCH1* (9q22.3)](http://www.ncbi.nlm.nih.gov/gtr/genes/5727/) | *STX1A* (7q11.23) |
| [*ATR* (3q22-q24)](http://www.ncbi.nlm.nih.gov/gtr/genes/545/) | [*FOXG1* (14q13)](http://www.ncbi.nlm.nih.gov/gtr/genes/2290/) | [*KCTD7*](http://www.cureepilepsy.org/egi/genes/kctd7.asp) (7q11.21) |  | *PTEN* (10q23.31) | [*STX1B*](http://www.cureepilepsy.org/egi/genes/stx1b.asp) (16p11.2) |
| [*BRD2* (6p21.3)](http://www.ncbi.nlm.nih.gov/gtr/genes/6046/) | [*FOXH1* (8q24.3)](http://www.ncbi.nlm.nih.gov/gtr/genes/8928/) | *KPNA7* (7q22.1) |  | *RS1* (Xp22.13) | [*SYN1* (Xp11.4-p11.2)](http://www.ncbi.nlm.nih.gov/gtr/genes/6853/) |
| [*CACNA1A* (19p13)](http://www.ncbi.nlm.nih.gov/gtr/genes/773/) | *FRRS1L* (9q31.3) | [*LBR* (1q42.1)](http://www.ncbi.nlm.nih.gov/gtr/genes/3930/) |  | [*RELN* (7q22)](http://www.ncbi.nlm.nih.gov/gtr/genes/5649/) | *SYNJ1* (21q22.11) |
| [*CACNA1H* (16p13.3)](http://www.ncbi.nlm.nih.gov/gtr/genes/8912/) | [*GABRA1* (5q34-q35)](http://www.ncbi.nlm.nih.gov/gtr/genes/2554/) | [*LGI1* (10q23.33)](http://www.ncbi.nlm.nih.gov/gtr/genes/9211/) |  | SCARB2 (4p21.1) | [*SYNGAP1*](http://www.cureepilepsy.org/egi/genes/syngap1.asp) (6p21.32) |
| [*CACNB4* (2q22-q23)](http://www.ncbi.nlm.nih.gov/gtr/genes/785/) | [*GABRB3* (15q11.2-q12)](http://www.ncbi.nlm.nih.gov/gtr/genes/2562/) | *LPCAT1* (5p15.33) |  | [*SCN10A* (3p24.2-p22)](http://www.ncbi.nlm.nih.gov/gtr/genes/6336/) | [*SZT2*](http://www.cureepilepsy.org/egi/genes/szt2.asp) (1p34.2) |
| [*CASK* (Xp11.4)](http://www.ncbi.nlm.nih.gov/gtr/genes/8573/) | [*GABRD* (1p36.3)](http://www.ncbi.nlm.nih.gov/gtr/genes/2563/) | [*MAGI2* (7q21)](http://www.ncbi.nlm.nih.gov/gtr/genes/9863/) |  | [*SCN11A* (3p24-p21)](http://www.ncbi.nlm.nih.gov/gtr/genes/11280/) | [*TBC1D24*](http://www.cureepilepsy.org/egi/genes/tbc1d24.asp) (16p13.3) |
| [*CASR* (3q13.3-q21)](http://www.ncbi.nlm.nih.gov/gtr/genes/846/) | [*GABRG2* (5q31.1-q33.1)](http://www.ncbi.nlm.nih.gov/gtr/genes/2566/) | [*MBD5* (2q23.1)](http://www.ncbi.nlm.nih.gov/gtr/genes/55777/) |  | [*SCN1A* (2q24)](http://www.ncbi.nlm.nih.gov/gtr/genes/6323/) | [*TCF4* (18q21.2)](http://www.ncbi.nlm.nih.gov/gtr/genes/6925/) |
| [*CCL2* (17q11.2-q12)](http://www.ncbi.nlm.nih.gov/gtr/genes/6347/) | [*GAMT* (19p13.3)](http://www.ncbi.nlm.nih.gov/gtr/genes/2593/) | [*MCPH1* (8p23)](http://www.ncbi.nlm.nih.gov/gtr/genes/79648/) |  | [*SCN1B* (19q13.1)](http://www.ncbi.nlm.nih.gov/gtr/genes/6324/) | [*TGIF1* (18p11.3)](http://www.ncbi.nlm.nih.gov/gtr/genes/7050/) |
| [*CDK5RAP2* (9q33.3)](http://www.ncbi.nlm.nih.gov/gtr/genes/55755/) | [*GATM* (15q15.3)](http://www.ncbi.nlm.nih.gov/gtr/genes/2628/) | [*ME2* (18q21)](http://www.ncbi.nlm.nih.gov/gtr/genes/4200/) |  | [*SCN2A*](http://www.cureepilepsy.org/egi/genes/scn2a.asp) (2p24.3) | [*TPP1* (11p15.5)](http://www.ncbi.nlm.nih.gov/gtr/genes/1200/) |
| [*CDKL5* (Xp22)](http://www.ncbi.nlm.nih.gov/gtr/genes/6792/) | *GFAP* (17q21.31) | [*MECP2* (Xq28)](http://www.ncbi.nlm.nih.gov/gtr/genes/4204/) |  | [*SCN2B* (11q23)](http://www.ncbi.nlm.nih.gov/gtr/genes/6327/) | *TRPM2* (21q22.3) |
| [*CDON* (11q24.2)](http://www.ncbi.nlm.nih.gov/gtr/genes/50937/) | [*GNAO1*](http://www.cureepilepsy.org/egi/genes/gnao1.asp) (16q13) | [*MEF2C* (5q14)](http://www.ncbi.nlm.nih.gov/gtr/genes/4208/) |  | [*SCN3A* (2q24)](http://www.ncbi.nlm.nih.gov/gtr/genes/6328/) | [*TSEN2* (3p25.1)](http://www.ncbi.nlm.nih.gov/gtr/genes/80746/) |
| [*CENPJ* (13q12.2)](http://www.ncbi.nlm.nih.gov/gtr/genes/55835/) | *GLDC* (9p24.1) | [*MFSD8*(4q28.1-q28.2)](http://www.ncbi.nlm.nih.gov/gtr/genes/256471/) |  | [*SCN3B* (11q23.3)](http://www.ncbi.nlm.nih.gov/gtr/genes/55800/) | [*TSEN34* (19q13.4)](http://www.ncbi.nlm.nih.gov/gtr/genes/79042/) |
| [*CEP152* (15q21.1)](http://www.ncbi.nlm.nih.gov/gtr/genes/22995/) | [*GLI2* (2q14)](http://www.ncbi.nlm.nih.gov/gtr/genes/2736/) | [*MTHFR* (1p36.3)](http://www.ncbi.nlm.nih.gov/gtr/genes/4524/) |  | [*SCN4A* (17q23.1-q25.3)](http://www.ncbi.nlm.nih.gov/gtr/genes/6329/) | [*TSEN54* (17q25.1)](http://www.ncbi.nlm.nih.gov/gtr/genes/283989/) |
| *CHD2* (15q26.1) | *GOSR2* (17q21.32) | *NCAM1* (11q23.2) |  | [*SCN4B* (11q23)](http://www.ncbi.nlm.nih.gov/gtr/genes/6330/) | [*UBE3A* (15q11-q13)](http://www.ncbi.nlm.nih.gov/gtr/genes/7337/) |
| [*CHRNA2* (8p21)](http://www.ncbi.nlm.nih.gov/gtr/genes/1135/) | *GUF1* (4p12) | *NECAP1* (12p13.31) |  | [*SCN5A* (3p21)](http://www.ncbi.nlm.nih.gov/gtr/genes/6331/) | [*VANGL1* (1p13)](http://www.ncbi.nlm.nih.gov/gtr/genes/81839/) |
| [*CHRNA4* (20q13.2-q13.3)](http://www.ncbi.nlm.nih.gov/gtr/genes/1137/) | [*GPR56* (16q13)](http://www.ncbi.nlm.nih.gov/gtr/genes/9289/) | [*NDE1* (16p13.1)](http://www.ncbi.nlm.nih.gov/gtr/genes/54820/) |  | [*SCN7A* (2q21-q23)](http://www.ncbi.nlm.nih.gov/gtr/genes/6332/) | [*WDR62* (19q13.12)](http://www.ncbi.nlm.nih.gov/gtr/genes/284403/) |
| [*CHRNB2* (1q21)](http://www.ncbi.nlm.nih.gov/gtr/genes/1141/) | [*GRIN1* (9q34.3)](http://www.ncbi.nlm.nih.gov/gtr/genes/2902/) | [*NDUFA1* (Xq24)](http://www.ncbi.nlm.nih.gov/gtr/genes/4694/) |  | [*SCN8A* (12q13)](http://www.ncbi.nlm.nih.gov/gtr/genes/6334/) | [*WWOX*](http://www.cureepilepsy.org/egi/genes/wwox.asp) (16q23.1-23.2) |
| [*CLCN2* (3q26)](http://www.ncbi.nlm.nih.gov/gtr/genes/1181/) | [*GRIN2A* (16p13)](http://www.ncbi.nlm.nih.gov/gtr/genes/2903/) | *NEDD4L* (18q21.31) |  | [*SCN9A* (2q24)](http://www.ncbi.nlm.nih.gov/gtr/genes/6335/) | [*ZEB2* (2q22)](http://www.ncbi.nlm.nih.gov/gtr/genes/9839/) |
| *CLCN4* (Xp22.2) | [*GRIN2B*](http://www.cureepilepsy.org/egi/genes/grin2b.asp) (12p13.1) | [*NF1* (17q11.2)](http://www.ncbi.nlm.nih.gov/gtr/genes/4763/) |  | [*SHH* (7q36)](http://www.ncbi.nlm.nih.gov/gtr/genes/6469/) | [*ZIC2* (13q32)](http://www.ncbi.nlm.nih.gov/gtr/genes/7546/) |
| [*CLN3* (16p12.1)](http://www.ncbi.nlm.nih.gov/gtr/genes/1201/) | [*HCN1* (5p12)](http://www.ncbi.nlm.nih.gov/gtr/genes/348980/) (5p12) | [*NHLRC1* (6p22.3)](http://www.ncbi.nlm.nih.gov/gtr/genes/378884/) |  | [*SIK1*](http://www.cureepilepsy.org/egi/genes/sik1.asp) (21q22.3) |  |
| [*CLN5* (13q21.1-q32)](http://www.ncbi.nlm.nih.gov/gtr/genes/1203/) | [*HCN3* (1q22)](http://www.ncbi.nlm.nih.gov/gtr/genes/57657/) | [*NODAL* (10q22.1)](http://www.ncbi.nlm.nih.gov/gtr/genes/4838/) |  | [*SIX3* (2p21)](http://www.ncbi.nlm.nih.gov/gtr/genes/6496/) |  |

Supplementary Table 2. The epileptic panel included 203 genes for patient 2.
